# Latent infection of *Vigna unguiculata* with seed-borne bean common mosaic virus modulates plant growth and may contribute to mutualistic symbiosis between the virus and host plant

**DOI:** 10.3389/fmicb.2025.1524787

**Published:** 2025-04-09

**Authors:** Hideki Takahashi, Nan Xu, Yoshinori Kanayama, Midori Tabara, Atsushi Takeda, Toshiyuki Fukuhara, Shuhei Miyashita

**Affiliations:** ^1^Graduate School of Agricultural Science, Tohoku University, Sendai, Japan; ^2^College of Life Sciences, Ritsumeikan University, Kusatsu, Japan; ^3^Department of Applied Biological Sciences and Institute of Global Innovation Research, Tokyo University of Agriculture and Technology, Tokyo, Japan

**Keywords:** bean common mosaic virus, beneficial effect, latent infection, mutualistic symbiosis, Potyviridae, virus seed transmission

## Abstract

In evaluating the germination and growth of the seed resources of 322 cultivars of cowpea (*Vigna unguiculata*), we found the development of yellow symptoms on ~50% of the cotyledons of 10 cultivars. RNA-Seq analysis of total RNA extracted from symptomatic cotyledons indicated that the 10 cultivars were infected with the bean common mosaic virus (*Potyvirus phaseovulgaris*, BCMV), which is a member of the family *Potyviridae* and able to seed-transmit to progeny plants. One of the BCMV isolates identified in the 10 cultivars was BCMV(Vu06), which was infected with cultivar #6. During the growth of BCMV(Vu06)-infected cowpea plants, there were no systemic symptoms in newly developing leaves, but the virus coat protein was detected in both leaves and flowers. Thus, the cowpea cultivar #6 plant was latently infected with BCMV(Vu06). There was no significant difference in the dry matter weight of the above-ground parts of the plant between BCMV(Vu06)-latently infected and non-infected plants. However, BCMV(Vu06)-latently infected plants had late flower and bud formation and longer life but slightly lower seed yield than the non-infected plants. The 1,000-seed weight and germination frequency of the seeds harvested from infected plants were the same as those of non-infected plants. Taken together, latent infection of cultivar #6 with BCMV(Vu6) modulates the balance between vegetative and reproductive plant growth and the longer lifespan of BCMV(Vu06)-latently infected plants may provide an advantage for its survivability over generations. BCMV(Vu06) and cowpea cultivar #6 might have established a mutual symbiotic relationship during their interaction.

## Introduction

Recent virome analysis of plants using next-generation sequencing (NGS) technology revealed that many plant species are sometimes infected with viruses without showing clear symptoms (Roossinck, 2015; Takahashi et al., 2019; Chofong et al., 2021). In particular, perennial plants that are persistently/latently infected with viruses appear to maintain the viruses throughout their life. Moreover, in the case of annual plants infected with attenuated strains of seed-borne viruses, the viruses can be transmitted to the next generation, although the frequency of seed transmission is influenced by a range of factors, including the virus strain and host cultivar (Khetarpal and Maury, 1987; Sastry, 2013; Worrall et al., 2015). Thus, annual plants are also able to maintain viruses without clear symptoms over host plant generations (Pagán, 2022).

A symbiotic interaction between viruses and host plants might enable the host plants to tolerate acute infection with related viruses, and one strategy used to control the occurrence of viral diseases is the latent infection of plants with a virus or attenuated virus (Nishiguchi and Kobayashi, 2011; Tomitaka et al., 2024). However, other biologically significant aspects of the commensal or mutualistic interaction between viruses and host plants have not been sufficiently investigated.

The bean common mosaic virus (*Potyvirus phaseovulgaris*, BCMV) is distributed worldwide and has a very high transmission rate through seed-borne spread, aphid vectors, and human factors such as agricultural operations (Worrall et al., 2015; Jordan and Hammond, 2021). BCMV is known for naturally infecting wild and crop legumes and has a typical viral structure as a member of the family *Potyviridae* (Worrall et al., 2015). The genome of BCMV comprises single-stranded RNA of approximately 10,000 nucleotides in length that encodes a polyprotein containing 10 mature proteins: P1, helper component-protease (HC-Pro), P3, 6 K1, cylindrical inclusion (CI), 6 K2, nuclease inclusion a (NIa)-viral genome-linked protein (VPg), NIa-Pro, nuclease inclusion b (NIb), and coat protein (CP) (Worrall et al., 2015; Jordan and Hammond, 2021). The existence of different pathogenic groups of BCMV was discovered in 1943, and the complex BCMV strains were subsequently classified into seven pathogenic groups (I–VII) based on the different symptoms of 10 bean cultivars (Drijfhout et al., 1978). Based on peptide profiles, the molecular weight of the CP, and genome size and sequence (Mckern et al., 1992), the taxonomy of BCMV has been reclassified as two serotypes, A and B, named BCMNV and BCMV, respectively (Berger et al., 1997). Furthermore, the deduced amino acid sequences of the CP and 3′-untranslated regions are currently registered on the NCBI database and can be used to classify BCMV isolates (Sharma et al., 2011).

Cowpea (*Vigna unguiculata*), which is one of the oldest crops in the history of human cultivation, is a host for BCMV (Singh et al., 1997). The symptoms of BCMV infection vary widely among the various combinations of cowpea cultivars and BCMV strains. However, the typical symptoms in BCMV-infected cowpea cultivars are light and dark mosaic patterns, leaf curling, dwarfing, and chlorosis (Jordan and Hammond, 2021). In BCMV-infected plants showing severe symptoms, the harvested seeds are clearly less numerous, smaller in shape, or malformed, and the seed yield is significantly reduced (Agrios, 2005). However, the severity of the symptoms and rates of seed transmission depend on the growth stage of the plant at the time of infection. Indeed, early infection of cowpea plants results in severe yield losses with many poorly growing seedlings, whereas the seed yield of plants infected later does not differ significantly from that of healthy plants (Morales and Castano, 1987; Galvez and Morales, 1989; Puttaraju et al., 2004). On the other hand, the latent infection of cowpea plants with BCMV or other seed-borne viruses has been reported (Odedara et al., 2009). However, the influence of BCMV latent infection on the life of cowpea plants is not investigated. In the view of better understanding the biological significance of the commensal or mutualistic interaction between viruses and host plants, extensive analysis of the life of cowpea plants latently infected with BCMV seems to provide new insight.

During a survey of the plant growth and morphology of 322 cultivars of cowpea [NARO GeneBank, National Agriculture and Food Research Organization (NARO), Tsukuba, Japan], we examined cultivars that were latently infected with BCMV but appeared to grow healthily. In this study, 10 BCMV isolates that had been used to infect 10 cultivars were characterized to clarify the taxonomical lineage of BCMV and to determine the influence of BCMV latent infection on the life of cowpea plants.

## Materials and methods

### Plants and virus

The seeds of 322 cowpea (*Vigna unguiculata*) cultivars were supplied by the public resource stock center NARO GeneBank (Supplementary Table S1). Seventeen seeds of each cultivar were sowed on soil and cultivated to observe symptom development. Of the 322 cultivars, 10 that exhibited vein yellowing or mosaic symptoms in their cotyledons were used for further analysis. These cultivars were named cultivars #1 to #11 for our current study due to the length of the original cultivar names in NARO GeneBank (Table 1 and Supplementary Table S1). The NARO GeneBank JP accession numbers of these 10 cultivars and their geographical origin are listed in Table 1. The cowpea seeds were sown in Metro-Mix® 380 soil (Sun Gro Horticulture, Agawam, MA, United States) and cultivated under a 14-h light (14,000 lx)/10-h dark photoperiod at 23°C in an LH-411S growth chamber (NK System Co. Ltd., Tokyo, Japan). Cowpea plants were used for the experiments 7 days after being sown.

**Table 1:** Detection of bean common mosaic virus (BCMV) or cucumber mosaic virus (CMV) in 10 cultivars of *Vigna unguiculata* supplied by NARO GeneBank.

GeneBank JP number	Altanative name of cultivar in this study^a^	Cultivar name	Origin	Collection year	Immunostrip	Symptoms at 20 days after sowing
85460	#1	COL/NEPAL/1984/IBPGR/I-638-3	Nepal	1986	BCMV+, CMV+	Severe
85463	#2	COL/NEPAL/1984/IBPGR/I-702-2	Nepal	1986	BCMV+, CMV+	Moderate
85471	#3	COL/NEPAL/1984/IBPGR/I-745	Nepal	1986	BCMV+, CMV+	Moderate
85443	#4	COL/NEPAL/1984/IBPGR/I-409-1	Nepal	1986	BCMV+, CMV−	No symptom
85453	#5	COL/NEPAL/1984/IBPGR/I-598	Nepal	1986	BCMV+, CMV−	No symptom
85429	#6	COL/NEPAL/1984/IBPGR/I-199	Nepal	1986	BCMV+, CMV−	No symptom
85427	#8	COL/NEPAL/1984/IBPGR/I-189	Nepal	1984	BCMV+, CMV−	Moderate
85407	#9	COL/NEPAL/1984/IBPGR/U-1247-1	Nepal	1986	BCMV+, CMV−	No symptom
85447	#10	COL/NEPAL/1984/IBPGR/I-423-1	Nepal	1984	BCMV+, CMV+	Very severe
85419	#11	COL/NEPAL/1984/IBPGR/U-1709-2	Nepal	1984	BCMV+, CMV+	Moderate

a In this study, alternative names were assigned to the cultivars due to the length of their original names.

Two BCMV strains [BCMV(Pn-F) (MAFF715049) and BCMV(12) (MAFF105007)] originating from Japan were also supplied by NARO GeneBank. The viruses were propagated using 4-week-old *Nicotiana benthamiana*, which was grown under the same conditions described above. Seven-day-old healthy cowpea plants were rub-inoculated with BCMV(Pn-F) or BCMV(12) using 600 mesh carborundum and then grown under the same conditions.

BCMV(Vu06) was purified from symptomatic leaves of BCMV(Vu06)-infected cultivar #6 by the standard method (Roh et al., 1996). BCMV-free cowpea cultivar #6 was rub-inoculated with the purified BCMV(Vu06) using 600 mesh carborundum and then grown under the same conditions.

### Immunological detection of the virus CP

To survey the BCMV infection of 322 cowpea cultivars, the CP of BCMV in the sap of each cowpea cotyledon, which was homogenized with 0.05 mM sodium phosphate buffer (pH 7.0), was immunologically detected using ImmunoStrip for Poty (Agdia, Elkhart, IN, United States) according to the instructions. Regarding BCMV-infected cotyledons, the CP of the cucumber mosaic virus (CMV) in their homogenates was also immunologically detected using ImmunoStrip for CMV (Agdia).

**Figure 1:**
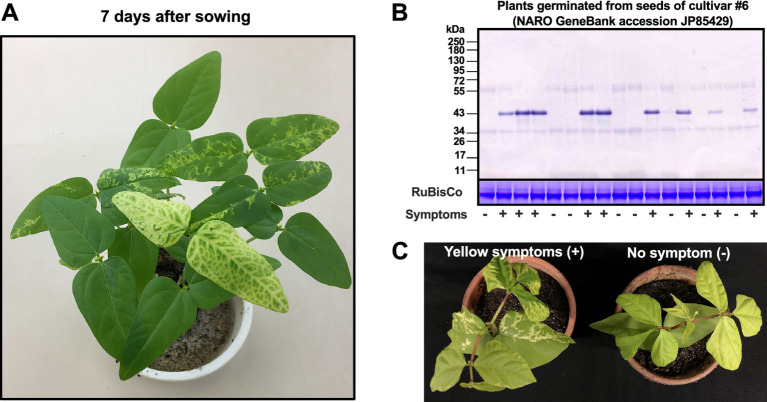
Symptom development and immunological detection of the coat protein of bean common mosaic virus strain Vu06 [BCMV(Vu06)] in the cotyledons of cowpea (*Vigna unguiculata*) cultivar #6. **(A)** Mosaic and vein yellowing symptoms in the cotyledons of cowpea plants at 7 days after sowing. **(B)** Immunological detection of the coat protein of BCMV(Vu06) in each cotyledon of plants showing yellow symptoms (+) or no symptoms (−). As an internal control, the band of RuBisCo is shown using CBB staining. **(C)** At 14 days after sowing, plants showing yellow symptoms and without symptoms were separately transferred to new pots.

To further analyze BCMV infection in cowpea nursery plants, two leaf disks with a 6 mm diameter were taken from the cotyledons of each plant 7 days after sowing or 7 days after BCMV(Pn-F) or BCMV(12) inoculation. To evaluate BCMV infection in mature cowpea plants at the vegetative stage, 10 leaf disks with a 6-mm diameter were randomly taken from 10 fully expanded leaves of each plant. To find BCMV infection in cowpea flowers, the petal, sepal, stigma, style, anther, filament, and ovary tissues were sampled from the flower using a blade (see Figure 1A). Each tissue sample was collected and weighed and then homogenized at five times its weight of GTEN buffer containing 25 mM Tris–HCl (pH 7.5), 1 mM EDTA (pH 8.0), 150 mM NaCl, and 10% glycerol using a plastic pestle on ice. The amount of total protein of each tissue for Western blot was determined using Bradford reagent (Bio-Rad, Hercules, CA, United States). The sample loading volume for SDS-PAGE was standardized based on the protein concentration. After the addition of an equal volume of sample buffer containing 125 mM Tris–HCl (pH 6.8), 4% SDS, 10% sucrose, 0.004% bromophenol blue, and 10% 2-mercapoethanol, the well-mixed homogenate was incubated at 98°C for 10 min and then centrifuged at 12,000 rpm for 5 min. A volume of 10 μL of each supernatant was applied to 12% SDS–polyacrylamide gel electrophoresis (SDS-PAGE). After electro-transferring the protein to a polyvinylidene difluoride membrane, the CP of BCMV was immunologically detected using an antibody against the CP of BCMV (Agdia, Elkhart, IN, United States). The SDS-PAGE, electro-transfer, and immunological detection procedures were conducted according to the standard protocol (Sambrook and Russell, 2001).

### Total RNA extraction and NGS analysis

Total RNA was extracted from each symptomatic cotyledon of 10 cowpea cultivars (#1, #2, #3, #4, #5, #6, #8, #9, #10, and #11) using an RNeasy Plant Mini Kit (QIAGEN, Hilden, Düsseldorf, Germany) according to the manufacturer’s instructions. An RNA sequencing library was prepared using a TruSeq Stranded Total RNA Sample Prep Kit (Illumina, San Diego, CA, United States) according to the Low Sample Prepare protocol. The library concentration was determined using a Library Quantification Kit (TaKaRa Bio, Shiga, Japan). MiSeq v3 Reagent Kit (Illumina) was used to obtain 75-bp paired-end reads. The reads were *de novo* assembled with SPAdes software (ver. 3.15.5) under RNA mode, followed by a BLASTn (ver. 2.14.1) search to find BCMV contigs.

### Determination of 5′- and 3’-terminal nucleotide sequences of BCMV RNA by the RACE method

To determine the 5′- and 3′-terminal nucleotide sequences of BCMV RNA, we used total RNA extracted from symptomatic cotyledons using an RNeasy Plant Mini Kit (QIAGEN). 5′- and 3′-terminal cDNAs of BCMV were synthesized from total RNA using a SMARTer®RACE 5′/3’ Kit (Takara Bio). For the 5′ and 3’ cDNA synthesis reaction, BCMV 5′-specific primer: 5’-GATTACGCCA AGCTTGCTGTTTGATGGTGCTTTGCTGTTGA-3′ and BCMV 3′-specific primer: 5’-GATTACGCCAAGCTTGCAC AGATGAAGG CAGCAGCCCTCAGC-3′, were used, respectively. The BCMV 5′- or 3′-specific primer sequence was a conserved nucleotide sequence located at a ~500 bp distance from the 5′ or 3′ end of BCMV cDNA obtained from NGS analysis of total RNA isolated from symptomatic cotyledons of 10 cultivars. All experimental procedures were conducted according to the manufacturer’s instructions.

The resultant 5′ and 3’ cDNA fragments were directly sequenced by the Sanger sequencing method using a CEQ8000 Automated DNA sequencer (Beckman Coulter, Brea, CA, United States). Full-length nucleotide sequences of 10 BCMV isolates, which were used to infect the seeds of 10 cultivars of cowpea, were assembled and registered in NCBI/ENA/DDBJ. These 10 BCMV isolates were named BCMV(Vu01), BCMV(Vu02), BCMV(Vu03), BCMV(Vu04), BCMV(Vu05), BCMV(Vu06), BCMV(Vu08), BCMV(Vu09), BCMV(Vu10), and BCMV(Vu11).

### Phylogenetic analysis

Full-length nucleotide sequences of the 10 BCMV isolates were first subjected to recombination detection using RDP5 software^1^ together with full-length or nearly full-length genome sequences of BCMV strains/isolates obtained from the NCBI/ENA/DDBJ database (21 sequences, Supplementary Table S2). Recombination events supported by all seven detection methods [RDP (Martin and Rybicki, 2000), GENECONV (Padidam et al., 1999), BOOTSCAN (Martin et al., 2005), MAXCHI (Smith, 1992), CHIMAERA (Posada and Crandall, 2001), SISCAN (Gibbs et al., 2000), and 3SEQ (Lam et al., 2018)] in RDP5 software were considered to have occurred. After removing the eight recombinant candidates, the remaining 23 sequences were subjected to phylogenetic analysis together with three PVY sequences (GenBank FJ214726, KJ603225, and HQ912911) as an outgroup. MEGAX software^2^ was used for muscle alignment and evolutionary history inference by the Maximum-Likelihood method with the Hasegawa–Kishino–Yano model (Hasegawa et al., 1985; Kumar et al., 2016). The bootstrap test was run for 1,000 replicates.

For the phylogenetic analysis of CP nucleotide sequences, CP nucleotide sequences were obtained from the NCBI/ENA/DDBJ database by a BLASTn search using the CP-coding region sequence of BCMV RefSeq (NC003397.1) as a query. Sequences detected with more than 90% query coverage and with place of collection information were further extracted. The 235 sequences obtained were subjected to phylogenetic analysis together with the 10 CP-coding region sequences determined in the current study and a BSMNV sequence (GenBank U190287) as an outgroup. MEGAX was used for muscle alignment and evolutionary history inference by the maximum-likelihood method with the Tamura–Nei model (Tamura and Nei, 1993), and the bootstrap test was run for 100 replicates to draw a consensus tree at a 30% cutoff value.

### Evaluation of vegetative and reproductive plant growth and seed germination

The influence of BCMV(Vu06) infection on cowpea plant growth was analyzed according to the procedure shown in Supplementary Figure S1. Cowpea seeds harvested from BCMV (Vu06)-infected cowpea cultivar #6 were sown. At 7 days after sowing, the development of yellow symptoms in cotyledons was evaluated, and eight plants showing symptoms and eight plants not showing symptoms were separately transferred to new pots. At 20, 30, 45, and 90 days after sowing, the plant growth status was photographed. At 20 days after sowing, an SPAD value reflecting chlorophyll content in the leaves was measured. To measure the average SPAD value (±standard deviation), 10 leaves of BCMV(Vu06)-infected cowpea or non-infected cowpea were randomly picked. At 20 days after sowing, the CP of BCMV(Vu06) in the leaves of BCMV(Vu06)-infected cowpea or non-infected cowpea was immunologically detected. At 45 days after sowing, the average dry matter weight (±standard deviation) of the above-ground parts of four BCMV(Vu06)-infected plants and four non-infected plants was measured. These results were used to evaluate the vegetative plant growth of BCMV(Vu6)-infected and non-infected plants.

At 30 or 45 days after sowing, the average number of pods (±standard deviation) of BCMV (Vu06)-infected or non-infected cowpea plants was counted. At 120 days after sowing, the average number (±standard deviation) of all seeds produced by four BCMV(Vu06)-infected or four non-infected cowpea plants was counted separately after pod maturity. To determine the average 1,000-seed weight (±standard deviation) of four BCMV(Vu06)-infected or four non-infected cowpea plants, 1,000 dried seeds were weighed. The results were used to evaluate the reproductive plant growth of BCMV(Vu6)-infected and non-infected plants.

The optimal temperature for the germination of cowpea seed is ~32°C (Barros et al., 2020). To evaluate the germination of the seeds harvested from BCMV(Vu6)-infected and non-infected plants, the seeds were sown on soil and incubated at 25°C or 35°C. The germination ratio was evaluated by counting the number of germinated seedlings at 7 days after sowing. At the same time, the average SPAD value (±standard deviation) of 10 cotyledons per plant showing yellow symptoms was measured. The experiments for evaluating plant growth and seed germination were conducted three times, and representative data are shown.

### Statistical analysis

The average ± standard deviation (SD) of SPAD value, plant dry weight, number of pods and seeds, 1,000-seed weight, and germination rate, which were obtained from independent BCMV(Vu06)-infected plants or independent non-infected control plants, was calculated. For statistical analysis, the comparison between two groups, BCMV(Vu06)-infected and non-infected control plants, was subjected to Welch’s *t*-test (*p* < 0.05). The horizontal line in each bar chart indicated the comparison between BCMV(Vu06)-infected and non-infected control plants. A significant difference between BCMV(Vu06)-infected and non-infected control plants was shown by the asterisk in the bar chart.

## Results

### Survey of virus infection in 322 cultivars of cowpea (*Vigna unguiculata*)

The plant growth and morphology of 322 cultivars of cowpea were evaluated 7 days after sowing. For the evaluation, 17 seeds of each cultivar were sowed on soil and cultivated. While 312 cultivars did not exhibit any symptoms (data not shown), vein yellowing or mosaic symptoms developed in the cotyledons of approximately 45% of nursery plants of each 10 cultivars, which were re-named cultivars #1, #2, #3, #4, #5, #6, #8, #9, #10, and #11 (Table 1). A survey of BCMV and CMV infection in the symptomatic cotyledons of these 10 cultivars using “ImmunoStrip” indicated that cultivars #1, #2, #3, #10, and #11 were mix-infected with BCMV and CMV, whereas cultivars #4, #5, #6, #8, and #9 were only infected with BCMV (Table 1). During the growing of cultivars #4, #5, #6, #8, and #9 (infected with BCMV alone), the appearance of systemic symptoms on newly developing leaves was attenuated, and no systemic symptoms were observed at 20 days after sowing (Table 1). On the other hand, at 20 days after sowing, the systemic symptoms in cultivars #1, #2, #3, #10, and #11 (mix-infected with BCMV and CMV) comprised severe or moderate yellowing (Table 1). Thus, the appearance of systemic yellowing symptoms seemed to be correlated with mix-infection with two viruses, and latent seed-borne infection with BCMV might occur in cultivars #4, #5, #6, #8, and #9.

### Characterization of 10 BCMV isolates transmitted through the seeds of 10 cultivars

To explore the origin of the BCMV isolates that were used to infect the seeds of 10 cultivars, the nucleotide sequences of the genomic RNA of 10 BCMV isolates were determined by NGS and the 5’-RACE and 3’-RACE method followed by Sanger sequencing using total RNA isolated from the cotyledons showing yellow symptoms. The 10 isolates of BCMV that were used to infect the seeds of the 10 cultivars were named Vu01, Vu02, Vu03, Vu04, Vu05, Vu06, Vu08, Vu09, Vu10, and Vu11. The nucleotide sequences of the genome RNA of the BCMV isolates, BCMV(Vu01), BCMV(Vu02), BCMV(Vu03), BCMV(Vu04), BCMV(Vu05), BCMV(Vu06), BCMV(Vu08), BCMV(Vu09), BCMV(Vu10), and BCMV(Vu11), were registered in the public database NCBI/ENA/DDBJ and assigned the accession numbers LC848290, LC848291, LC848292, LC848293, LC848294, LC848295, LC848296, LC848297, LC848298, and LC848299, respectively. The deduced amino acid sequences of the polyprotein encoded in the genomic RNA of the 10 BCMV isolates had 91–96% homology to three reference BCMV strains (GenBank accession numbers KC832501, AJ312438, and NC_003397) (Supplementary Table S2).

### Recombination detection and phylogenetic analysis of 10 BCMV isolates based on complete nucleotide sequences of viral genomic RNA

Out of 21 full-length or nearly full-length BCMV genome sequences in the NCBI/ENA/DDBJ database (Supplementary Table S3) and the 10 full-length nucleotide sequences of BCMV isolates determined in this study, eight [BCMV(Vu08), BCMV(US-10), BCMV(NL-4), BCMV(Y), BCMV(PV0915), BCMV(RU-1), BCMV(Soybean), and BCMV(Taiwan)] were considered recombinants in the RDP5 analysis. Thus, the remaining 23 sequences were used to draw a phylogenetic tree, together with three PVY sequences as an outgroup (Supplementary Figure S2). The analyses showed that the BCMV isolates obtained in this study formed two clades, one comprising BCMV(Vu01) and BCMV(Vu02) and the other comprising BCMV(Vu03), BCMV(Vu04), BCMV(Vu05), BCMV(Vu06), BCMV(Vu09), BCMV(Vu10), and BCMV(Vu11). Both clades belonged to a larger clade of BCMV isolates collected in East Asia (Supplementary Figure S2).

A phylogenetic tree for the CP-coding region was also drawn. From all of the BCMV CP nucleotide sequences registered in the NCBI/ENA/DDBJ database, 235 sequences with isolation origin information were extracted (Supplementary Table S5) and were used for phylogenetic analysis together with the 10 CP-coding sequences of BCMV isolates determined in the current study and a BCMNV CP sequence as an outgroup (Supplementary Figure S3). The generated tree exhibited three main clades, one comprising isolates originating from China (red), one comprising isolates from East, Southeast, and South Asia (green), and one comprising isolates from Non-East or Southeast Asia (blue). Regarding the CP sequences of the 10 BCMV isolates obtained in this study, all 10 sequences belonged to the East, Southeast, and South Asia clades (i.e., the green clade in Supplementary Figure S3). However, no clear similarity to South Asian (i.e., Indian) isolates was detected. Therefore, we considered that it is difficult to determine whether these 10 BCMV isolates originated from Nepal or Japan.

### Influence of BCMV(Vu06) infection on the growth of cowpea plants

When BCMV(Vu06)-free cultivar #6 was inoculated with purified BCMV(Vu06), mild mosaic symptoms only developed on non-inoculated upper leaves at the early growth stage of the inoculated plant (Supplementary Figure S4A), and after that, as the plant grew, the symptom was attenuated (data not shown). Half of the nursery plants, which were germinated from the seeds harvested from purified BCMV(Vu06)-latently infected cultivar #6, exhibited yellowing or mosaic symptoms on the cotyledon (Supplementary Figure S4B). BCMV coat protein was detected from symptomatic leaves but not non-symptomatic leaves of the germinated plants at the next generation (Supplementary Figure S4C). The results suggested that BCMV(Vu06) can latently infect cultivar #6 and be seed-transmitted to the next generation. Therefore, to study the role of latent infection with BCMV on the life of cowpea plant, we focused on the combination of BCMV(Vu06) and cowpea cultivar #6 as representative of cowpea latently infected with BCMV through seeds. Figure 1A shows nursery plants of cultivar #6 at 7 days after sowing. The accumulation of the 32-kDa CP of BCMV(Vu06) was immunologically detected in plants showing vein yellowing or mosaic symptoms in their cotyledons but not in symptomless plants (Figure 1B). Thus, the correlation between BCMV(Vu06) and yellow symptoms in the cotyledons of cultivar #6 was reconfirmed. To further analyze BCMV(Vu06)-infected and non-infected plants, eight nursery plants exhibiting yellow symptoms in their cotyledons and eight symptomless nursery plants were separately transferred to a new pot (Figure 1C) and further cultivated in the phytotron.

**Figure 2:**
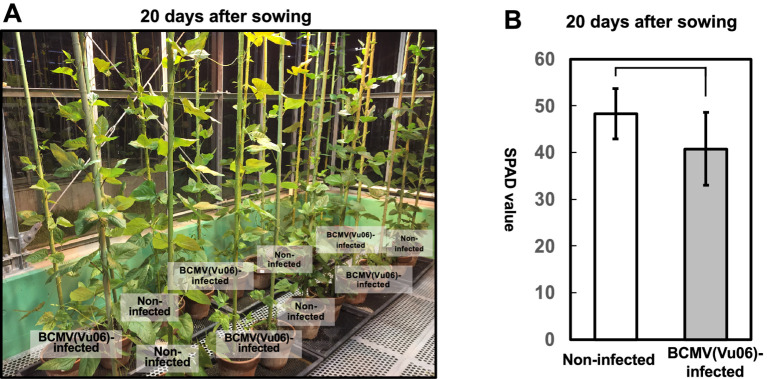
Comparison of plant growth of bean common mosaic virus strain Vu06 [BCMV(Vu06)]-infected and non-infected cowpea cultivar #6 at 20 days after sowing. **(A)** Photograph of BCMV(Vu06)-infected and non-infected cowpea cultivar #6 plants at 20 days after sowing. **(B)** SPAD value reflecting chlorophyll contents of the leaves of BCMV(Vu06)-infected and non-infected cowpea cultivar #6 at 20 days after sowing. The average SPAD value is shown in a bar chart with error bars (*n* = 8, SD). Statistical analysis did not denote significant differences between BCMV(Vu06)-latently infected and non-infected cowpea plants (Welch’s *t*-test, *p* < 0.05).

**Figure 3:**
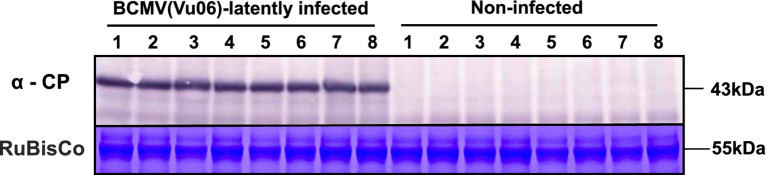
Immunological detection of the coat protein (CP) of bean common mosaic virus strain Vu06 [BCMV(Vu06)] in the leaves of cowpea cultivar #6 plants at 20 days after sowing. Eight plants exhibited yellow symptoms in their cotyledons [BCMV(Vu06)-latently infected] and another eight plants did not exhibit any symptoms in their cotyledons [non-infected]. As an internal control, the band of RuBisCo is shown using CBB staining.

**Figure 4:**
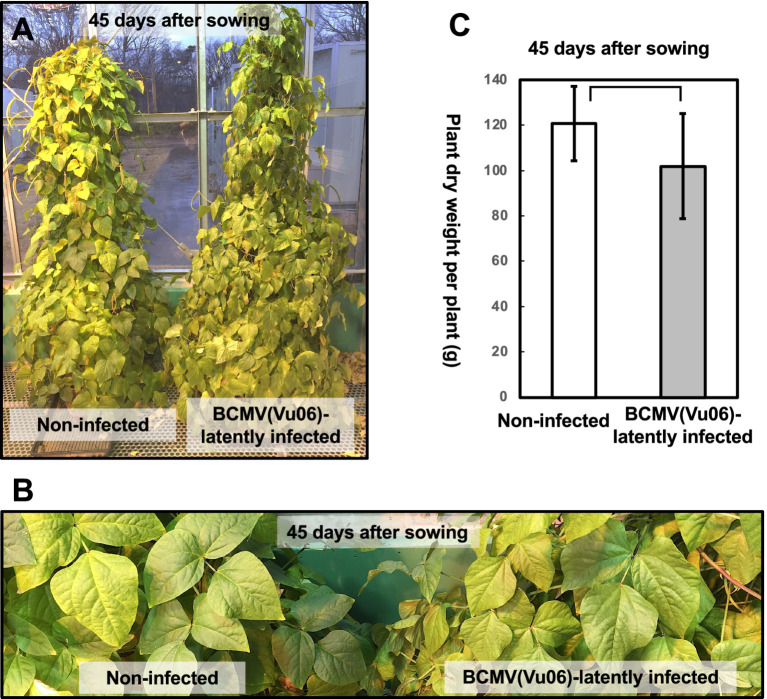
Comparison of plant growth of bean common mosaic virus strain Vu06 [BCMV(Vu06)]-infected and non-infected cowpea cultivar #6 at 45 days after sowing. **(A)** Photograph of a representative plant latently infected with BCMV(Vu06) or non-infected plant. **(B)** Enlarged photograph of the leaves of plants latently infected with BCMV(Vu06) or non-infected plant. **(C)** Dry material weight of the above-ground parts of BCMV(Vu06)-latently infected or non-infected cowpea cultivar #6 plants at 45 days after sowing. The average of dry material weight of the above-ground part was shown by a bar chart with error bars (*n* = 4, SD). Statistical analysis did not denote significant differences between BCMV(Vu06)-latently infected and non-infected cowpea plants (Welch’s *t*-test, *p* < 0.05).

During plant cultivation, systemic symptoms were attenuated in cultivar #6 infected with BCMV(Vu06), and no systemic symptoms were observed 20 days after sowing (Figure 2A). Furthermore, there was no significant difference in the SPAD value reflecting chlorophyll content between BCMV(Vu06)-infected and non-infected plants (Figure 2B), suggesting that yellow symptoms did not develop in the leaves of either plant group at 20 days after sowing. However, 20 days after sowing, the systemic spread of BCMV(Vu06) was confirmed in the symptomless BCMV(Vu06)-infected plants by immunoblotting (Figure 3). Furthermore, systemic symptoms were not observed on BCMV(Vu06)-infected plants at 30 and 45 days after sowing as well as non-infected plants (Supplementary Figure S5 and Figure 4B). Thus, the latent infection of the cowpea plant with BCMV occurred in the combination of cultivar #6 and BCMV(Vu06).

During the further cultivation of BCMV(Vu06)-latently infected or non-infected plants in the phytotron, there was no difference in plant growth at the vegetative stage (45 days after sowing) between BCMV(Vu06)-latently infected or non-infected cowpea plants (Figure 4A). Moreover, the dry material weight of the above-ground parts of the plants, whose pods were removed and harvested 45 days after sowing, was not significantly different between BCMV(Vu06)-latently infected plants and non-infected plants (Figure 4C). Thus, latent infection with BCMV(Vu06) did not influence the vegetative growth of cowpea cultivar #6.

### Influence of BCMV(Vu06) latent infection on pod formation in cowpea plants

**Figure 5:**
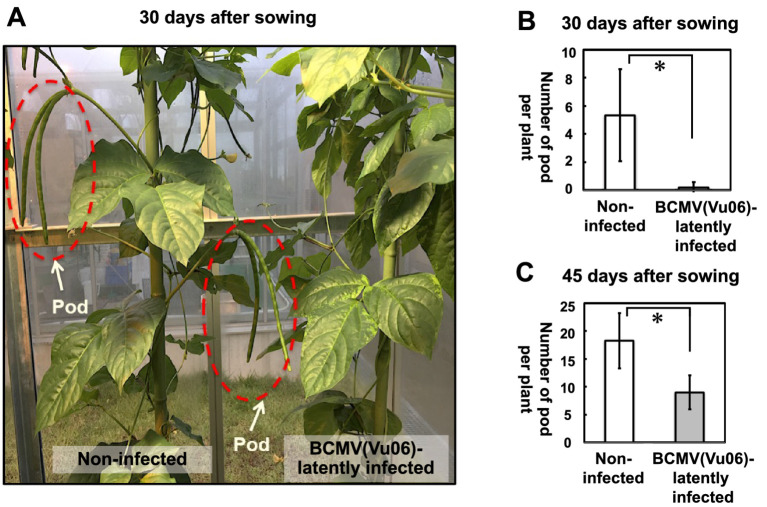
Measurement of the average number of the pods in bean common mosaic virus strain Vu06 [BCMV(Vu06)]-latently infected or non-infected cowpea plants at 30 or 45 days after sowing. **(A)** Photograph of pod formation in BCMV(Vu06)-latently infected or non-infected cowpea plants at 30 days after sowing. The pod is marked by a red dotted line. **(B)** Number of pods from BCMV(Vu06)-latently infected or non-infected cowpea plants at 30 days after sowing. **(C)** Number of pods from BCMV(Vu06)-latently infected or non-infected cowpea plants at 45 days after sowing. The average number of pods at 30 days **(B)** of 45 days **(C)** after sowing was shown by a bar chart with error bars (*n* = 4, SD). Statistical analysis denoted significant differences between BCMV(Vu06)-latently infected and non-infected cowpea plants by the asterisk (Welch’s *t*-test, *p* < 0.05).

At 30 days after sowing, non-infected plants began to produce a pod. However, in most BCMV(Vu06)-latently infected cowpea cultivar #6 plants, no pod formation was observed (Figure 5A). At 30 days after sowing, the average number of pods of non-infected plants was eight pods per plant, while the number of pods of BCMV(Vu06)-latently infected plants was nearly none (Figure 5B). In addition, at 45 days after sowing, the average number of pods of non-infected cowpea plants was approximately 18 pods per plant, while the average number of pods of BCMV(Vu06)-latently infected cowpea plants was less than 10 pods per plant (Figures 4A, 5C). However, the number of seeds in one pod of a BCMV(Vu06)-latently infected plant was very similar to that of a non-infected plant (data not shown). Significant differences in the number of pods in the same period indicated that non-infected plants might bear pods earlier than BCMV(Vu06)-latently infected plants.

### Influence of BCMV(Vu06) latent infection on the life of the cowpea plant

**Figure 6:**
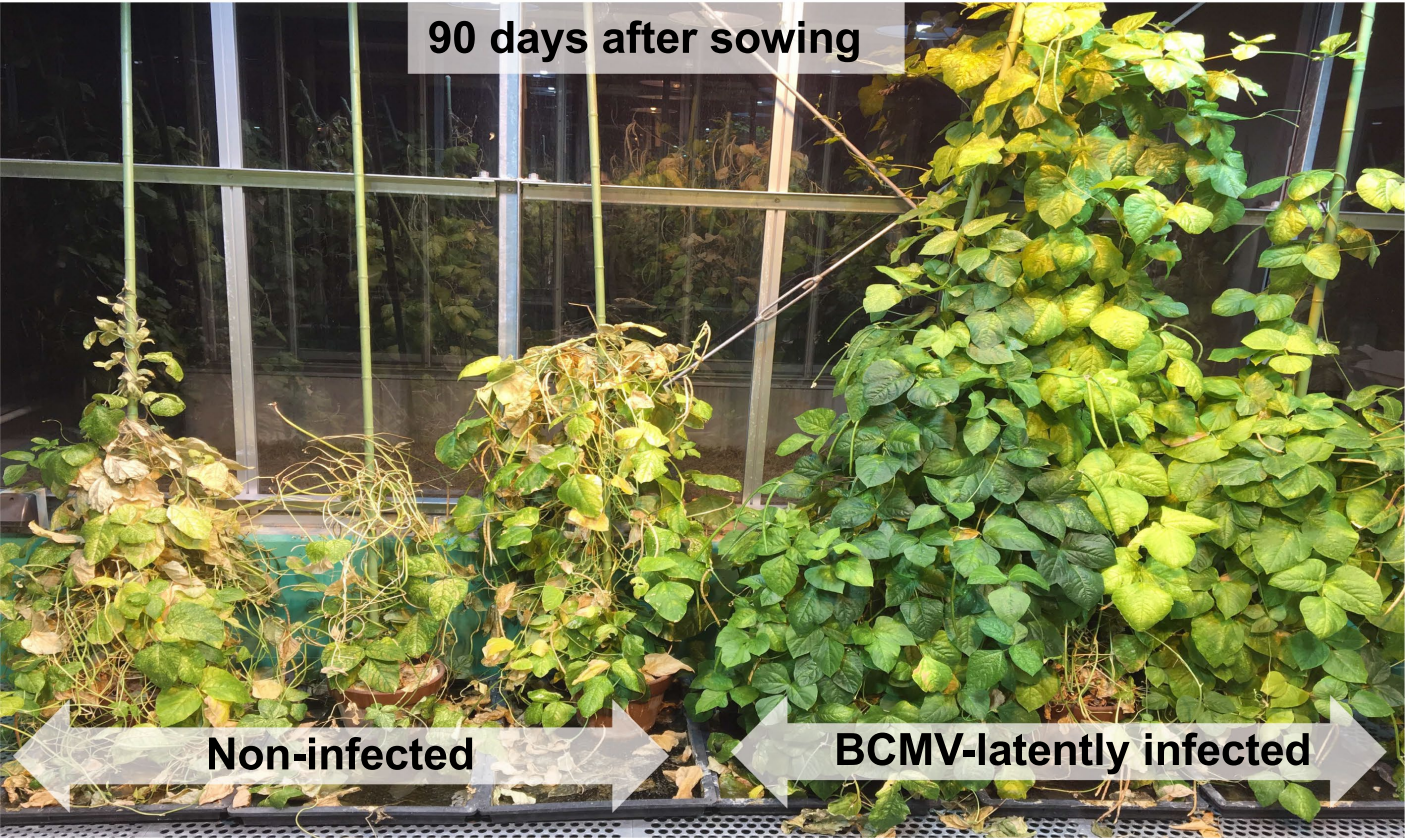
Growth of bean common mosaic virus strain Vu06 [BCMV(Vu06)]-latently infected or non-infected cowpea cultivar #6 plants at 90 days after sowing.

At 90 days after sowing, the upper part of non-infected cowpea plants had wilted due to senescence, while the upper part of BCMV(Vu06)-latently infected cowpea plants remained green and continued to grow (Figure 6). This result suggests that non-infected cowpea cultivar #6 plants mature and senescence faster than plants latently infected with BCMV(Vu06). The results demonstrated that BCMV(Vu06) latent infection seemed to delay the senescence of cowpea cultivar #6 plants.

### Influence of BCMV(Vu06) latent infection on the seed production of cowpea plants

**Figure 7:**
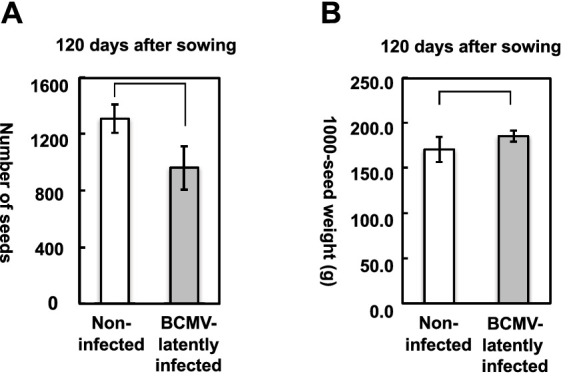
Measurement of seed production in bean common mosaic virus strain Vu06 [BCMV(Vu06)]-latently infected or non-infected cowpea plants at 120 days after sowing. **(A)** Number of seeds of BCMV(Vu06)-latently infected or non-infected cowpea plants. **(B)** Thousand-seed weight of BCMV(Vu06)-latently infected or non-infected cowpea plants. The average of the number of seeds **(A)** or 1,000-seed weight **(B)** was shown by a bar chart with error bars (*n* = 4, SD). Statistical analysis did not denote significant differences between BCMV(Vu06)-latently infected and non-infected cowpea plants (Welch’s *t*-test, *p* < 0.05).

Mature seeds of BCMV(Vu06)-latently infected or non-infected cowpea plants were collected separately from the mature pods at 120 days after sowing. The average number of seeds of non-infected cowpea plants was more than 1,200 seeds per plant, while the average number of seeds of BCMV(Vu06)-latently infected cowpea plants was approximately 900 seeds per plant (Figure 7A). Thus, cowpea plants latently infected with BCMV(Vu06) produced ~25% fewer seeds per plant than non-infected plants during their life.

After the harvested seeds were dried, the 1,000-seed weight of seeds collected from BCMV(Vu06)-latently infected cowpea plant was very similar to that of the non-infected plant (Figure 7B). Despite the fact that more seeds were harvested in the reproductive stage from non-infected cowpea plants, the seeds of BCMV(Vu06)-latently infected or non-infected cowpea plants had a similar 1,000-seed weight, indicating that BCMV(Vu06)-latently infected cowpea plants might be able to produce seeds as well as non-infected cowpea plants.

### Virus distribution in BCMV(Vu06)-latently infected flower tissue and comparison of seed transmission rates among BCMV strains

BCMV(Vu06) was transmitted to the next generation through seeds at a rate of ~50% (Figure 1A). When the cotyledons of non-infected cowpea cultivar #6 were inoculated with two BCMV strains [BCMV(Pn-F) and BCMV(12)] originating from Japan and supplied by NARO GeneBank, the CPs of the two BCMV strains were detected in each inoculated cotyledon (Supplementary Figure S6), and systemic symptoms developed in the mature stage of both virus-inoculated plants (data not shown). However, when 16 seeds that were harvested from BCMV(Pn-F)-infected or BCMV(12)-infected cowpea cultivar #6 were sown, no germinated seedlings showed yellowing symptoms (data not shown). Moreover, the CP was not detected in any seedling (Supplementary Figure S7). Thus, seed-borne transmission with latent infection with BCMV in cowpea seems to be established in the specific combination of cowpea cultivar #6 and BCMV(Vu06) and is not general to cowpea and BCMV.

**Figure 8:**
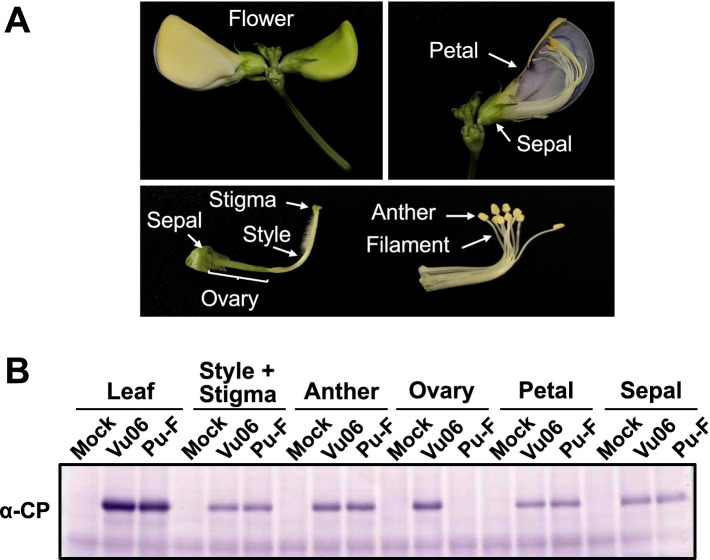
Detection of bean common mosaic virus (BCMV) coat protein (CP) in flower tissues of BCMV-latently infected cowpea plants. **(A)** Photograph of the structure of flower tissues of cowpea plant. **(B)** Immunological detection of the CP of BCMV(Vu06) and BCMV(Pn-F) in the petal, sepal, stigma, style, anther, filament, and ovary. Quantification of protein by the Bradford method as an internal reference. BCMV-infected leaves (Leaf) and each tissue of a healthy cowpea plant (Mock) were used as positive and negative controls.

To confirm BCMV transmission via seeds, the virus distribution in BCMV-latently infected flower tissue was analyzed by immunological detection of the CP. Figure 8A shows the structure of the flower, including the petal, sepal, stigma, style, anther, filament, and ovary. In BCMV(Vu06)-latently infected cowpea plant, the CP accumulated in all tissues of the flower, except the ovary (Figure 8B). However, the CP could not be detected in the ovary but was detected in other tissues of BCMV(Pn-F)-infected cowpea plant (Figure 8B). Thus, seed transmission of BCMV(Vu06) might be caused by infection of the ovary tissue of cultivar #6 with BCMV(Vu06).

### Influence of BCMV(Vu06) latent infection on the seed germination rate and symptom development at different temperatures

**Figure 9:**
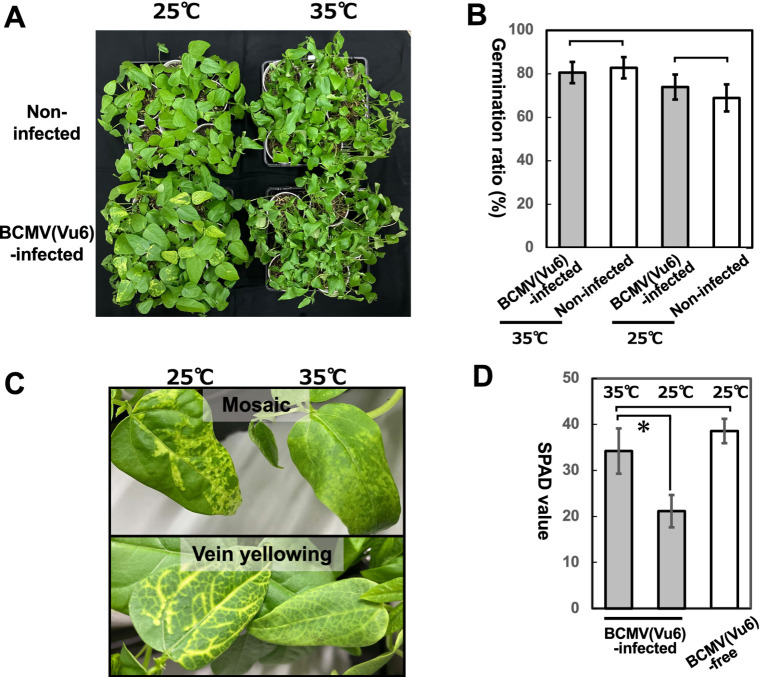
Germination ratio of seeds harvested from cowpea cultivar #6 latently infected with bean common mosaic virus strain Vu06 [BCMV(Vu06)] or non-infected plants. **(A)** Photograph of nursery plants germinated from seeds harvested from cowpea cultivar #6 latently infected with BCMV(Vu06) or non-infected plants at 25°C and 35°C. **(B)** Germination ratio of seeds harvested from cowpea cultivar #6 latently infected with BCMV(Vu06) or non-infected plants at 25°C and 35°C. **(C)** Photograph of mosaic or vein yellowing symptoms that developed in the cotyledons of germinated plants at 25°C and 35°C. **(D)** SPAD value reflecting chlorophyll content in symptomatic cotyledons of germinated plants at 25°C and 35°C. The average of the germination ratio of seeds **(B)** or SPAD value **(D)** was shown by a bar chart with error bars (*n* = 10, SD). Statistical analysis did not denote significant differences in the germination ratio of seeds **(B)** but significant differences in the SPAD value **(D)** between BCMV(Vu06)-latently infected and non-infected cowpea plants by the asterisk (Welch’s *t*-test, *p* < 0.05).

The germination rate and symptom development in the cotyledon at 25°C and 35°C were compared between the seeds harvested from BCMV(Vu06)-latently infected and non-infected seeds (Figure 9A). At 7 days after sowing at 35°C, the germination rate of the seeds harvested from BCMV(Vu06)-latently infected and non-infected plants was significantly higher than at 25°C (Figure 9B). In addition, the severities of the vein yellowing and mosaic symptoms that developed on BCMV(Vu06)-latently infected cotyledon at 35°C were attenuated in comparison with 25°C (Figure 9C). This symptom attenuation was quantitatively supported by measuring the SPAD value of BCMV(Vu06)-latently infected cotyledon (Figure 9D). Under 25°C and 35°C conditions, there was no difference in the germination rate of the harvested seeds between BCMV(Vu06)-latently infected and non-infected plants (Figure 9B). These results indicated that latent infection with BCMV(Vu06) did not influence the germination potential of the harvested seeds.

## Discussion

### Characterization of 10 BCMV isolates transmitted through cowpea seeds

BCMV is one of the causal agents of severe diseases in cowpea (*Vigna unguiculata*), which is one of the more widely cultivated legumes in the world (Morales, 2006). On the survey of 322 cultivars of cowpea, seed-borne infection with 10 BCMV isolates, which were named BCMV(Vu01), BCMV(Vu02), BCMV(Vu03), BCMV(Vu04), BCMV(Vu05), BCMV(Vu06), BCMV(Vu08), BCMV(Vu09), BCMV(Vu10), and BCMV(Vu11), was found in 10 cultivars that exhibited yellow symptoms on their cotyledons at 7 days after sowing. The complete nucleotide sequences of the genomic RNA of the 10 BCMV isolates were determined, and the amino acid sequences of the polyprotein encoded by the 10 viral genomic RNAs were highly homologous to the BCMV reference strains registered in the NCBI/ENA/DDBJ database (Supplementary Table S2). Although phylogenetic analysis of the amino acid sequences of the polyproteins encoded by BCMV suggested that the BCMV isolate formed two independent clades that were close to a larger clade of East Asia (Supplementary Figure S2), phylogenetic analysis using a large number of amino acid sequences encoding the CP region of BCMV indicated that it was difficult to determine the specific country of origin of the BCMV isolates (Supplementary Figure S3). This was because the CP amino acid sequences of the BCMV isolates did not show a high geographical homology to the amino acid sequences of BCMV strains isolated from specific countries. Moreover, there is no record of the amino acid sequence of the complete polyprotein or the CP encoded in the genomic RNA of BCMV isolated from Nepal in the database. Therefore, further information on the nucleotide sequences of BCMV RNA, including BCMV isolated from Nepal, may clarify whether the 10 seed-borne BCMV isolates originated in Nepal.

Regarding 312 cultivars that did not have any symptoms, there is a possibility that some cultivars were latently infected with BCMV because the immunological detection method may not have enough sensitivity to detect a lower amount of virus coat protein. Therefore, further analysis of virus latent infection in 312 cultivars by qPCR of RNA-seq seems to be necessary. Meanwhile, 312 cultivars may contain a cultivar showing resistance to BCMV. Thus, the screening of BCMV-resistant cultivars from 312 cultivars appears to be another interesting topic.

### Impact of BCMV(Vu06)-latent infection on the life of cowpea cultivar #6

Reports indicating that latent infection with viruses has beneficial effects on the life of the host plant are accumulating (Roossinck, 2015; Takahashi et al., 2019; Chofong et al., 2021). For example, latent infection with CMV confers host plants with drought and cold tolerance, alteration of pollinator preference, tolerance to deterioration, and change in the balance of root development (Bueso et al., 2017; Xu et al., 2008; Mauck et al., 2010; Westwood et al., 2013; Groen et al., 2016; Tabara et al., 2021, 2024; Takahashi et al., 2022). Adaptation of *Arabidopsis halleri* to an excess zinc environment is accelerated by latent infection with CMV (Tabara et al., 2024). Moreover, latent infection with double-stranded RNA viruses classified as *Amalgaviridae*, *Endornaviridae*, and *Partitiviridae* influences beneficial physiological traits, host plant growth, and the behavior of aphids, which are a vector of virus transmission (Xie et al., 1994; Nakatsukasa-Akune et al., 2005; Khankhum and Valverde, 2018; Fukuhara et al., 2020; Safari et al., 2019). Regarding *Potyviridae*, latent infection of cucurbitaceous plant with zucchini yellow mosaic virus (ZYMV) reduces susceptibility to fungal pathogens and attraction to cucumber beetle as a vector for ZYMV (Shapiro et al., 2013; Harth et al., 2018). Thus, our finding that the modulation of the balance of vegetative and reproductive plant growth by latent infection of cowpea (*Vigna unguiculata*) cultivar #6 with BCMV(Vu06) seems to provide new knowledge for understanding the role of latent virus infection in the life of host plants.

The frequency of virus transmission from BCMV-infected legume plants to their progeny generally varies between 0% and ~50% and is affected by a range of factors, including host cultivar., virus strain, time of infection, and environmental conditions (Morales and Castano, 1987; Galvez and Morales, 1989; Puttaraju et al., 2004). The seed transmission rate of BCMV(Vu06) in cowpea cultivar #6 was determined to be approximately 56.3%, while the other two BCMV strains, BCMV(Pn-F) and BCMV(12), which originated in Japan, were not transmitted through the seeds in cultivar #6. One of the reasons for the difference in the seed transmission of BCMV in cultivar #6 seems to be the ability of BCMV(Vu06) to infect the ovary of cultivar #6. This is because the accumulation of BCMV CP was detected in the ovary of the seeds harvested from BCMV(Vu06)-latently infected cultivar #6 but not BCMV(Pn-F)-infected cultivar #6, even though the CP was detected in other tissues of the flower of both BCMV-infected plants (Figure 8B). The ovary is an essential part of the reproductive organ of the flower. After fertilization and ripening, the ovules inside the ovary develop to become the seeds of cowpea plants. Thus, the ability of BCVMV(Vu06) to infect the ovary in the flower of cultivar #6 may allow it to be transmitted to the progeny through the seeds.

**Figure 10:**
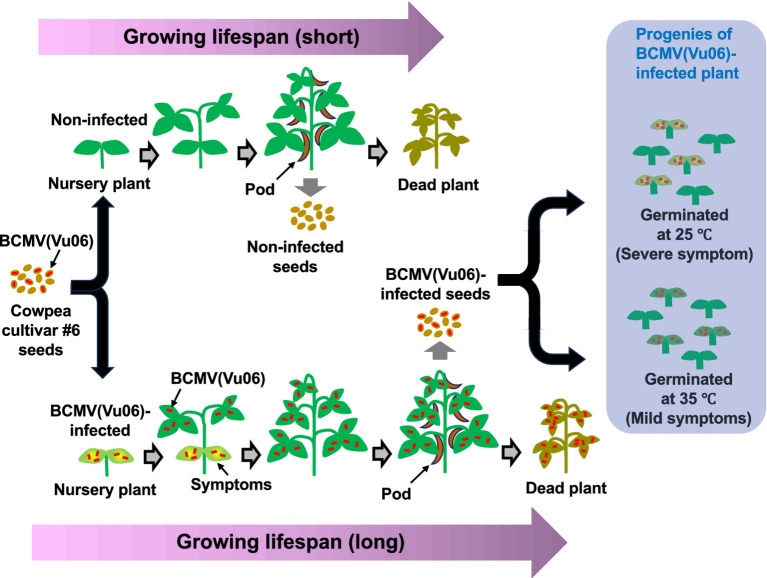
Schematic diagram of the growth and seed production of bean common mosaic virus strain Vu06 [BCMV(Vu06)]-latently infected and non-infected cowpea cultivar #6 plants throughout their growing phase. During growing, the appearance of systemic symptoms in newly developing leaves was clearly attenuated, although the virus coat protein was detected in those leaves and flowers as well as in the cotyledons. There was no significant difference in the dry matter weight of the above-ground parts of the plant between BCMV(Vu06)-latently infected and non-infected plants. BCMV(Vu06)-latently infected plants had late flower and bud formation and a long life, in comparison with non-infected plants. The 1,000-seed weight of the infected plants was the same as that of non-infected plants, and the germination frequency of the seeds harvested from BCMV-latently infected plants was also the same as that of non-infected plants.

The influence of BCMV(Vu06) latent infection on the life of cowpea cultivar #6 plants is summarized in an illustration shown in Figure 10. The infection of cowpea cultivar #6 seed with BCMV(Vu06) was easily distinguished by the development of yellow symptoms on the cotyledon at 7 days after sowing. However, during the growth of the infected plant, while systemic symptoms were obviously attenuated, the virus systemically spread without symptoms and reached the flower tissue of cowpea plants. The lifespan of cowpea cultivar #6 latently infected with BCMV(Vu06) was clearly longer than that of the non-infected plant. Although the yield of seeds harvested from cowpea plants latently infected with BCMV(Vu06) was slightly lower than that of non-infected healthy cowpea plants, the 1,000-seed weight of the seeds harvested from BCMV(Vu06)-latently infected plants was the same as that of non-infected plants. Moreover, there was no difference in the germination rate of the seeds between BCMV(Vu06)-latently infected and non-infected plants. In agricultural production systems, a higher yield of cowpea seeds during a short cultivation period would be a beneficial trait. However, in nature, the long lifespan of BCMV(Vu06)-latently infected cowpea cultivar #6 with mature seed production and stable transmission of the virus over generations may provide a survival advantage for the cowpea plant. According to this perspective, the interaction between BCMV(Vu06) and cowpea cultivar #6 may establish mutualistic symbiosis. The mutualistic symbiosis of BCMV(Vu06) and cowpea cultivar #6 provides new insight into the role of viruses in the life of host plants in nature.

## Data Availability

The NCBI/ENA/DDBJ accession numbers for cDNAs to BCMV(Vu01), BCMV(Vu02), BCMV(Vu03), BCMV(Vu04), BCMV(Vu05), BCMV(Vu06), BCMV(Vu08), BCMV(Vu09), BCMV(Vu10), and BCMV(Vu11) are LC848290, LC848291, LC848292, LC848293, LC848294, LC848295, LC848296, LC848297, LC848298, and LC848299, respectively.

## Footnotes

^1^ http://web.cbio.uct.ac.za/~darren/rdp.html

^2^ https://www.megasoftware.net/
