# Supplementary material for: Latent infection of Vigna unguiculata with seed-borne bean common mosaic virus modulates plant growth and may contribute to mutualistic symbiosis between the virus and host plant
Source: Front Microbiol. 2025 Apr 9;16:1524787. doi: 10.3389/fmicb.2025.1524787 (PMC12015941; doi:10.3389/fmicb.2025.1524787)
Supplement: Supplementary file 1 [file Data_Sheet_1.pdf]

Online Supplementary Material

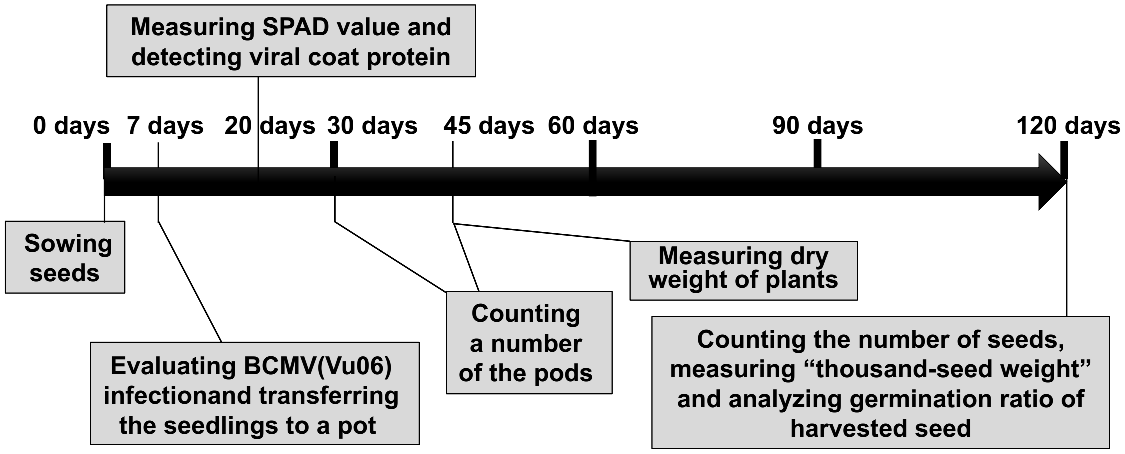

Figure S1. Schematic diagram of experimental procedure at each stage of cowpea plants growth and development. Bean common mosaic virus strain Vu06 [BCMV(Vu06)] infection was evaluated at 7 days after sowing. At 20, 30 and 90 days after sowing, the status of plant growth was photographed. SPAD value reflecting chlorophyll content was measured and viral coat protein was detected immunologically at 20 days after sowing. The number of pods was counted at 30 and 45 days after sowing, and the number of seeds and thousand-seed weight were analyzed at 120 days after sowing.

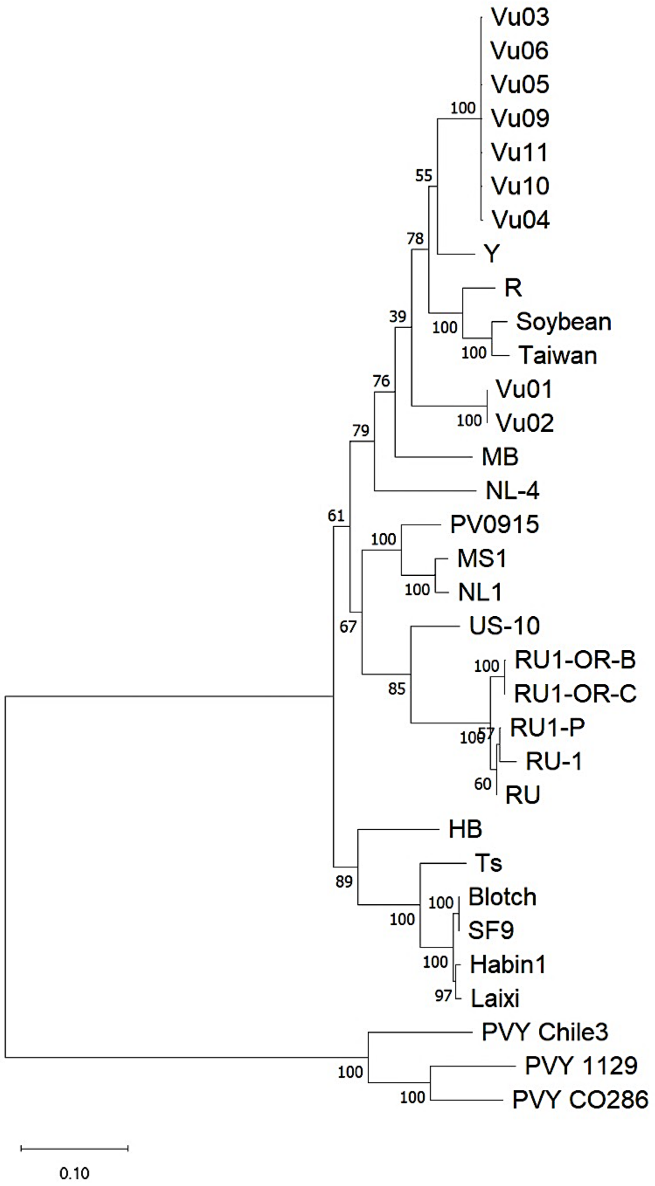

Figure S2. Phylogenetic tree of full-length nucleotide sequences of bean common mosaic virus (BCMV) genomic RNA (Vu01, Vu02, Vu03, Vu04, Vu05, Vu06, Vu08, Vu10 and Vu11), by running MEGAX with the Maximum-Likelihood method and Hasegawa-Kishino-Yano model. The bootstrap test was run for 1,000 replicates. 15 sequences of BCMV and three PVY sequences as an outgroup, which were available through NCBI database, were used for the analysis.

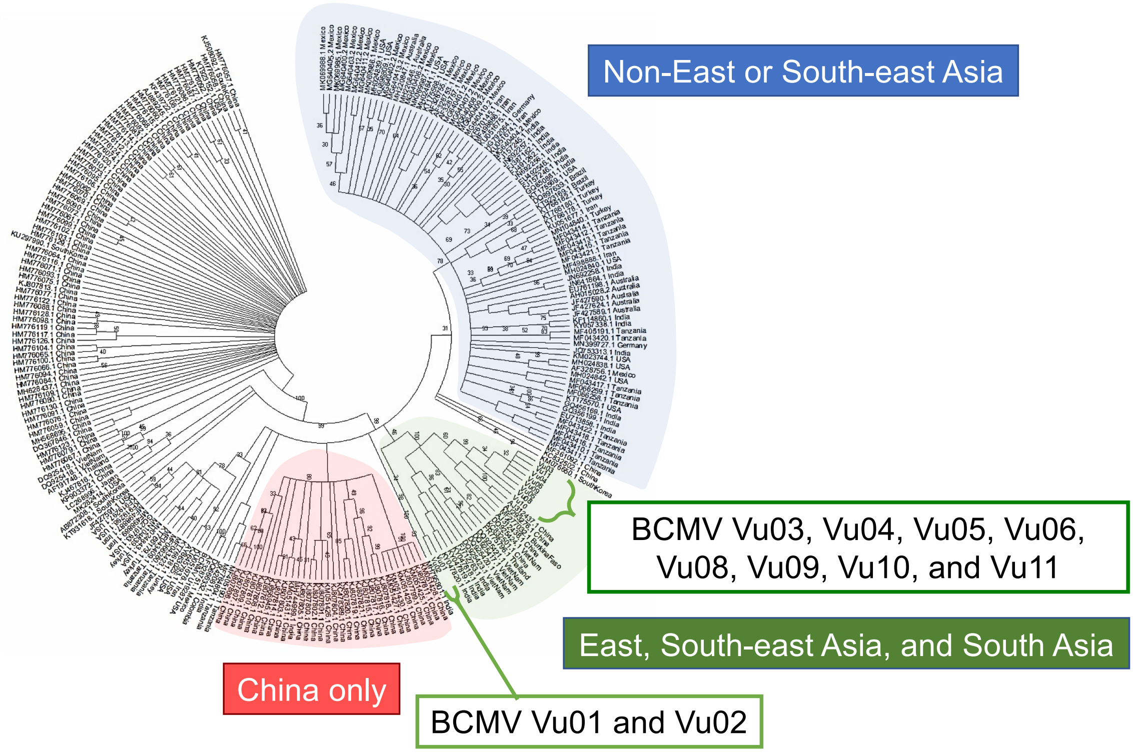

Figure S3. Phylogenetic tree of bean common mosaic virus (BCMV) nucleotide sequences of coat protein region, by running MEGAX with the Maximum-Likelihood method and Tamura-Nei model. The bootstrap test was run for 100 replicates. 235 sequences and a BSMNV sequence as an outgroup, which were available through NCBI database, were used for the analysis. The red region means that the sequences are originated from China. The green region means that the sequences are done from East, South-east, and South Asia. The blue region means that the sequences are done from Non-East or South-east Asia.

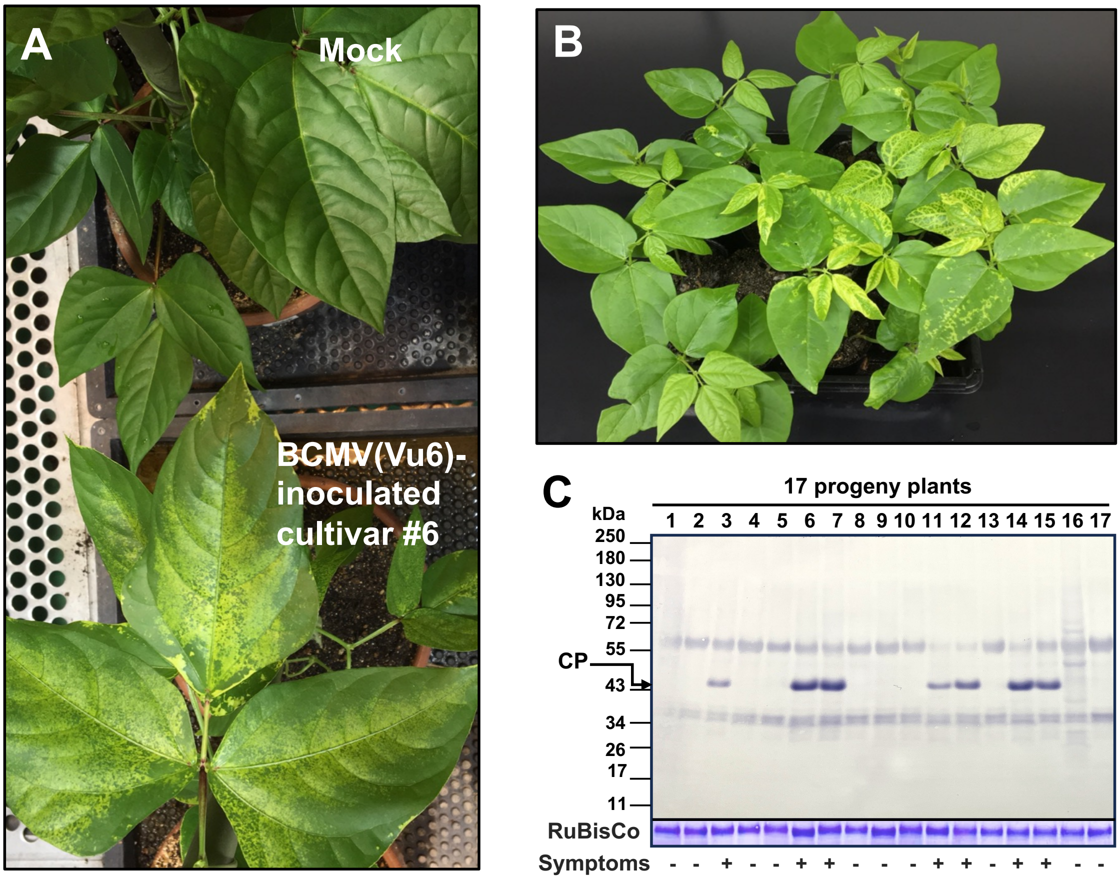

Figure S4. Bean common mosaic virus strain Vu06 [BCMV(Vu06)] infection on virus-free cowpea (*Vigna unguiculata*) cultivar #6 inoculated with BCMV(Vu06) and seed-transmission of BCMV(Vu06) to progeny plants. (**A**) Development of mild symptoms on non-inoculated upper leaves at 14 days after virus inoculation. Primary leaves of virus-free cultivar #6 was inoculated with purified BCMV(Vu06). (**B**) Photograph of nursery progeny plants germinated from the seeds harvested from BCMV(Vu06)-infected cultivar #6. (**C**) Immunological detection of the coat protein (CP) in each cotyledon of the 17 individual progeny plants showing yellow symptoms (+) or no symptoms (-). As an internal control, the band of RuBisCo is shown using CBB staining.

30 days after sowing

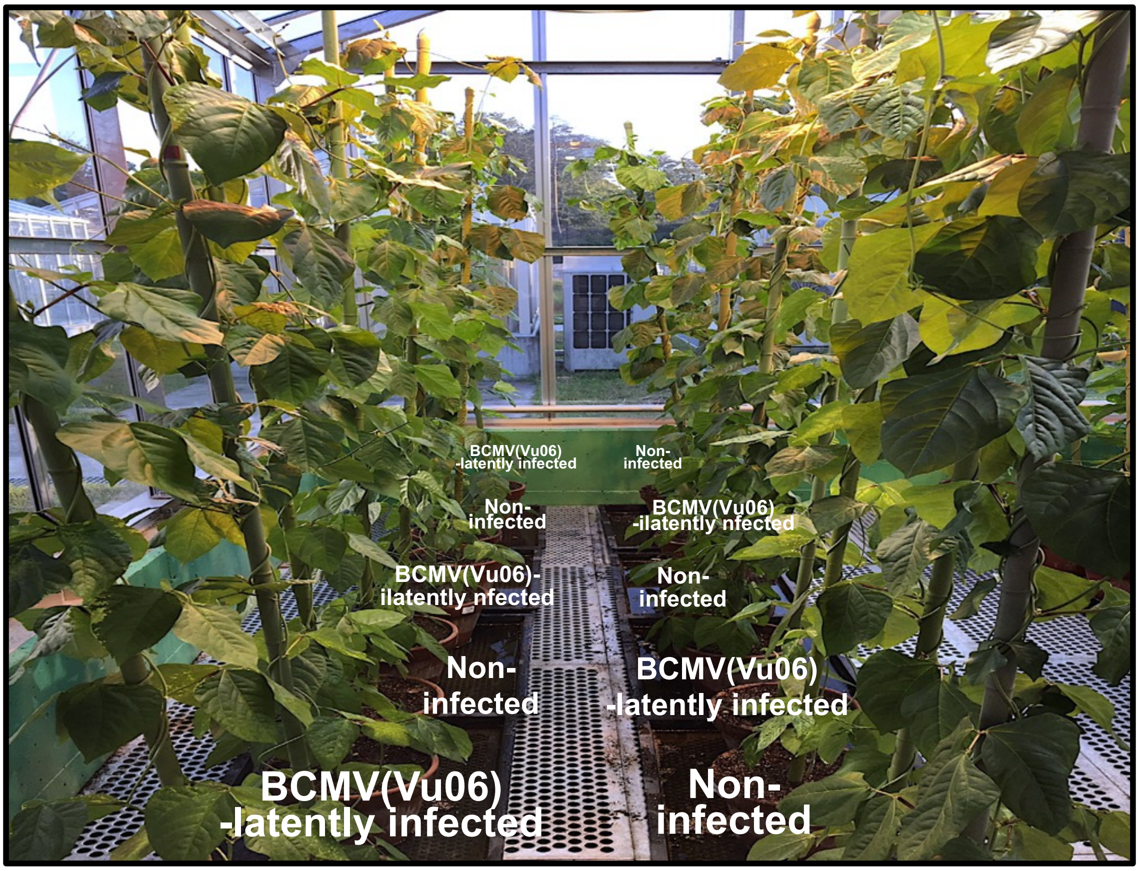

Figure S5. Comparison of bean common mosaic virus strain Vu06 [BCMV(Vu06)]-latently infected and non-infected cowpea cultivar #6 at 30 days after sowing.

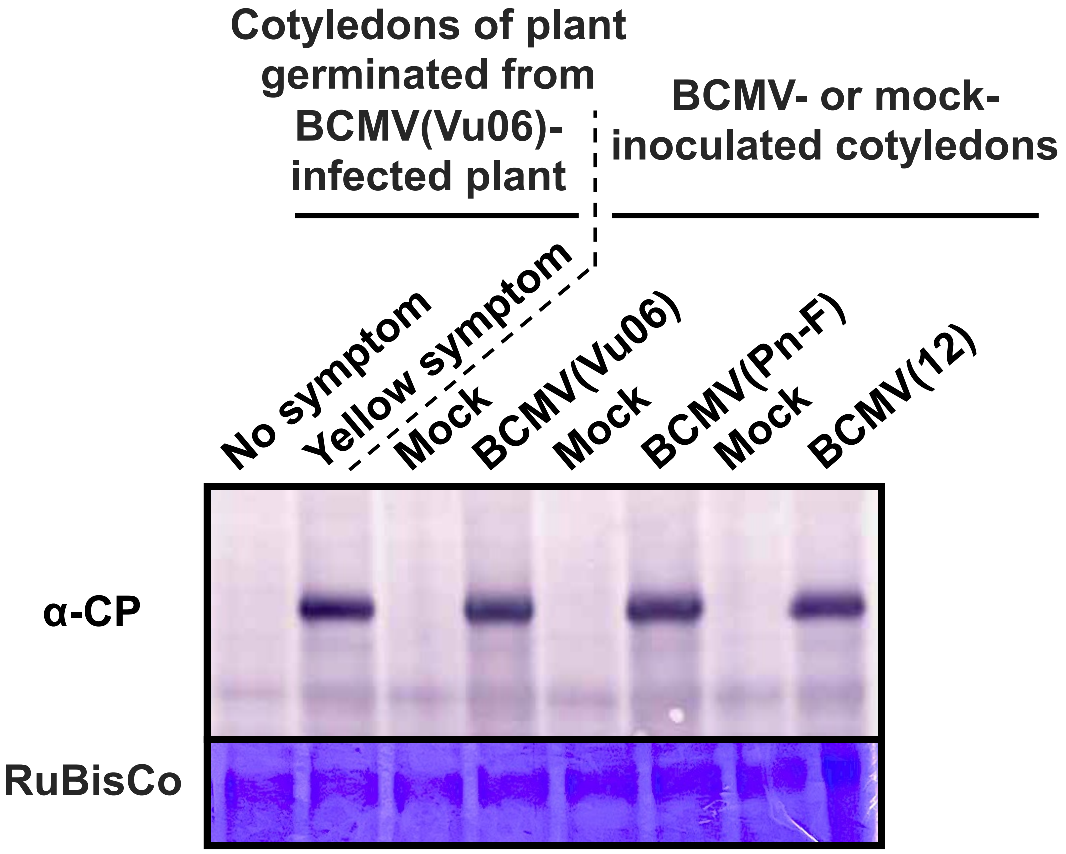

Figure S6. Immunological detection of the coat protein of bean common mosaic virus (BCMV) in the cotyledons of cowpea cultivar #6 which were inoculated with BCMV(Vu06), BCMV(Pn-F) and BCMV(12), respectively. RuBisCo was shown as an internal reference for protein quantity. As a control, the samples taken from the cotyledons showing yellow symptom and no symptom, which were germinated from BCMV(Vu06)-infected plant, were applied on the gel. As an internal control, the band of RiBisCo was shown by CBB staining.

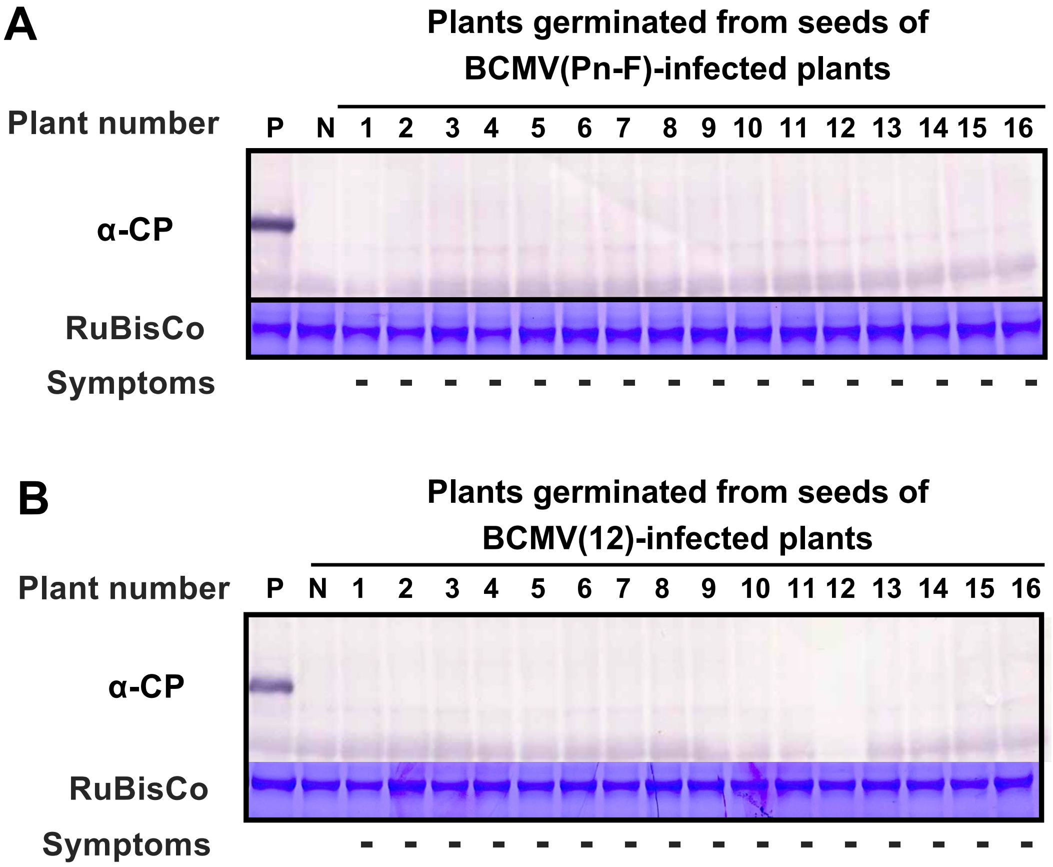

Figure S7. Immunological detection of the coat protein of bean common mosaic virus (BCMV) in the cotyledons of nursery plants which were germinated from the seeds harvested from BCMV(Pn-F)- or BCMV(12)-infected cowpea cultivar #6 plants, respectively. (**A**) BCMV(Pn-F)-infected cowpea cultivar #6 plants. (**B**) BCMV(12)-infected cowpea cultivar #6 plants. RuBisCo protein was shown as an internal reference for protein quantity. BCMV-infected leaves (P) and mock-infected leaves (N) were used as positive and negative control. As an internal control, the band of RiBisCo was shown by CBB staining.
